# Fertility in Advanced Societies: A Review of Research: La fécondité dans les sociétés avancées: un examen des recherches

**DOI:** 10.1007/s10680-012-9277-y

**Published:** 2012-09-12

**Authors:** Nicoletta Balbo, Francesco C. Billari, Melinda Mills

**Affiliations:** 1 Department of Sociology (ICS), University of Groningen, Groningen, The Netherlands; 2 Department of Sociology, Nuffield College, University of Oxford, Oxford, UK

**Keywords:** Fertility tempo, Postponement, Fertility quantum, Low fertility, Determinants of fertility, Calendrier de la fécondité, Report de la procréation, Intensité de la fécondité, Basse fécondité, Déterminants de la fécondité

## Abstract

This paper provides a review of fertility research in advanced societies, societies in which birth control is the default option. The central aim is to provide a comprehensive review that summarizes how contemporary research has explained ongoing and expected fertility changes across time and space (i.e., cross- and within-country heterogeneity). A secondary aim is to provide an analytical synthesis of the core determinants of fertility, grouping them within the analytical level in which they operate. Determinants are positioned at the individual and/or couple level (micro-level), social relationships and social networks (meso-level); and, by cultural and institutional settings (macro-level). The focus is both on the quantum and on the tempo of fertility, with a particular focus on the postponement of childbearing. The review incorporates both theoretical and empirical contributions, with attention placed on empirically tested research and whether results support or falsify existing theoretical expectations. Attention is also devoted to causality and endogeneity issues. The paper concludes with an outline of the current challenges and opportunities for future research.

## Résumé

Cet article présente un aperçu des recherches dans le domaine de la fécondité réalisées dans les sociétés dites avancées, c’est-à-dire les sociétés dans lesquelles le contrôle des naissances est l’option par défaut. L’objectif principal est de fournir une vue d’ensemble complète résumant comment la recherche contemporaine explique les changements de fécondité actuelle et prévue dans le temps et dans l’espace (c’est-à-dire l’hétérogénéité à l’intérieur d’un pays ou entre pays). Un second objectif vise à fournir une synthèse des principaux déterminants de la fécondité en les regroupant par niveau d’analyse dans lequel ils se situent. Les déterminants sont ainsi situés au niveau individuel ou au niveau du couple (niveau micro), au niveau des relations sociales et des réseaux sociaux (niveaux méso) et au niveau des cadres institutionnels et culturels (niveau macro). L’accent est mis tant sur l’intensité que le calendrier de la fécondité avec un intérêt particulier sur le report de la procréation. Cette synthèse de la littérature concerne les recherches tant théoriques qu’empiriques, une attention particulière étant portée à celles qui sont testées empiriquement et dont les résultats confirment ou infirment les théories explicatives existantes. De même nous nous sommes particulièrement intéressés aux problèmes de causalité et d’endogénéité. En conclusion, un tableau des défis actuels et des perspectives futures en matière de recherche est esquissé.

## Introduction

Fertility has been a central topic of research within the discipline of demography, but has also achieved considerable interest within sociology, anthropology, economics, medicine and psychology. During the last two decades, research about fertility in advanced societies—societies in which birth control is the default option—has flourished. It is not surprising, therefore, that several reviews of the existing fertility literature have been undertaken (Hirschman 1994; Caldwell and Schindlmayr 2003; Sobotka 2004; Butler 2004; Morgan and Taylor 2006; Mills et al. 2011). These reviews have provided important insights (although sometimes focusing on specific disciplines or geographical areas), while simultaneously outlining potential directions for future research. Since the 1980s, an increasing number of European and Asian countries reached very low fertility levels (Caldwell and Schindlmayr 2003) and virtually all advanced societies witnessed a ‘postponement’ transition (Kohler et al. 2002a, b). For this reason, the topic of low fertility dominated fertility research in advanced societies. During the late 2000s, a reversal of the fertility decline in most advanced countries—albeit with great heterogeneity—has drawn considerable attention (Goldstein et al. 2009; Myrskyla et al. 2009; OECD 2011). Furthermore, the impact of economic uncertainty and the recent economic recession on fertility is another emerging topic (Mills and Blossfeld 2005; Kreyenfeld 2010; Sobotka et al. 2011).

The central and overarching question of this study is: What is the state of fertility research today and where is it going? To achieve this goal, our review first classifies existing studies according to the determinants of fertility. We then ask: To what extent can we make use of the determinants of fertility described in existing research to explain ongoing and expected fertility changes over time and space (i.e., cross-country and within-country heterogeneity in fertility levels)? A secondary aim is to provide an analytical synthesis of the core determinants of fertility, grouping them within the analytical level at which they operate. Determinants are positioned at the micro-level, including determinants at the individual and/or couple level; the meso-level, which encompasses social relationships and social networks (i.e., characteristics pertaining to the network of friends, coworkers, relatives and/or to the relationship that links them to the individual); at the macro-level of the cultural and institutional settings where individuals and couples are embedded. This article not only provides a contemporary review of research and delineation of determinants, but extends our knowledge by adopting an analytical strategy to categorize these determinants, with the aim to provide a better understanding of the often highly interdisciplinary and complex task of explaining fertility trends and differences.

We likewise extend existing reviews by adopting a broader multidisciplinary approach, which takes into account relevant contributions from different disciplines beyond those that have been examined previously (i.e., often demography and sociology). We also embrace the most contemporary advances in the field, with attention to the recent fertility reversal in advanced societies. We acknowledge that the topic is highly interdisciplinary, with the term ‘fertility’ often taking on different meanings across disciplines. Subject areas are also diverse with research that examines non-human and non-animal fertility, mainly within the biological and environmental sciences. We focus only on human fertility within advanced societies, and cover research primarily within the disciplines of demography, sociology, medicine, biochemistry, genetics, molecular biology, economics and econometrics, psychology, decision and health sciences.

The present study reviews existing fertility research in a systematic and comprehensive manner, by looking at the two components that constitute human fertility: *tempo* (i.e., the timing of childbirth) and *quantum* (i.e., the total number of children). When examining *tempo*, we know that OECD countries have witnessed a rising mean age at first birth since the 1970s, coupled with an increasing proportion of births among mothers at advanced ages, albeit with considerable country-level variation (Billari et al. 2007; Sobotka et al. 2010; Mills et al. 2011). This process is generally referred to as the postponement of childbearing, which is the central focus of tempo studies in fertility research. The emergence of a ‘postponement transition’ of higher ages at first birth is a core phenomenon that has emerged in the last decades (Kohler et al. 2002a, b; Billingsley 2010).

*Quantum* is related to the number of children (including childlessness). While demographic transition theory implies that the quantum of fertility would stabilize around the replacement level of around 2.1 children per woman (e.g., Bongaarts 2002), during the last decades of the twentieth century, advanced societies witnessed low, or even the ‘lowest-low’ or ‘ultra-low’ fertility levels close to 1 (e.g., Kohler et al. 2002a, b; Frejka et al. 2010). The novelty of this phenomenon captivated demographers and raised the concerns of policy-makers about both the long-term demographic sustainability of their populations and concerns about the growing gap between desired and achieved fertility (Chesnais 1996; Bongaarts 2001; Goldstein et al. 2003; OECD 2011). Research has demonstrated that actual childbearing levels (i.e., the number of children born by the end of the reproductive age span) have not been as low as the standard quantum measure, the total fertility rate (TFR), would suggest (Bongaarts ad Feeney 1998; Kohler et al. 2002a, b; Sobotka 2004). Just as there appeared to be a consensus that advanced societies would either remain at low fertility levels or reach even lower levels (e.g., Lutz et al. 2003), new research produced evidence demonstrating the start of a fertility reversal during the 2000s (Goldstein et al. 2009; Myrskyla et al. 2009; OECD 2011).

Postponement is clearly interrelated with quantum since the age at first birth influences the (final) total number of children. Timing has always had an important influence on quantum, which is largely a measurement issue, since some of the measures that are used to study the quantum of fertility are not exclusively affected by changes in the timing of fertility. The most well-known case, for instance, is the TFR. Even at the micro-level there is still an influence of timing on quantum, since ‘postponement’ or earlier (perhaps unplanned) births are likely to affect the total number of children in some way. This is particularly relevant when studying the intended parity-progression over a certain time interval (a growing topic during the recent years). For these reasons, we will also highlight the interaction between tempo and quantum.

The remainder of this article is structured as follows. Section 2 focuses on the determinants of fertility at the micro-level, followed by Sect. 3, which investigates factors acting at the meso-level. Section 4 reviews fertility research at the macro-level. We conclude with a discussion that examines the current challenges and suggestions for future fertility research.

## Micro-Level Determinants of Fertility

Research at the micro-level focuses on the individual and/or couple decision-making process underlying the choice to have a child and investigates which circumstances affect decisions regarding the timing and number of children. Choices are often characterized as a rational response to uncertainty and/or as conformity to the prescribed ‘sequencing’ of life course events. A rich body of research has studied the link between life course circumstances and trajectories (mainly, partnership, education, employment and economic conditions) and fertility behavior. Other research has instead focused on the role of social class and family or origin, which in turn shapes an individual’s values and preferences. A major obstacle in this line of research is the challenge to establish causality, or in other words, the ability to empirically determine whether these life course factors are actual determinants of fertility or whether certain life course factors and fertility behavior are simultaneously affected by the presence of other common determinants (endogeneity or reverse causality).

### Role of Intentions in The Fertility Decision-Making Process

Many scholars have used *fertility intentions* as a proximate determinant for actual fertility behavior, examining which factors influence the formation, realization and/or change in fertility intentions (e.g., Westoff and Ryder 1977; Schoen et al. 1999; Quesnel-Vallée and Morgan 2003; Berrington 2004; Liefbroer 2009; Philipov 2009; Spéder and Kapitány 2009; Morgan and Rackin 2010; Iacovou and Tavares 2011). Others have identified the systematic gap between intended and actual fertility as one of the causes of low fertility (Morgan and Taylor 2006), since it reveals an ‘unmet need’ for children (Liefbroer 2009).

Spéder and Kapitány (2009) provide a detailed overview of the rich debate on the predictive power of fertility intentions on actual behavior. Next to critical approaches that downplay the explanatory power of intentions and less individual-specific measures such as ideal family size (e.g., Westoff and Ryder 1977; Quesnel-Vallée and Morgan 2003), other studies argue that intentions are effective predictors of actual fertility (Schoen et al. 1999; Berrington 2004). The majority of these latter studies draw upon the social-psychological literature, often employing the theory of planned behavior (TPB) (Ajzen 1991). The TPB has been explicitly adopted, among others, by for example, Billari et al. (2009) in a study of Bulgaria, Dommermuth et al. (2009) in Norway, as well as used to develop the questionnaires in the Generation and Gender Programme (Vikat et al. 2007). According to the TPB, intentions are the culmination of a combination of three antecedents: (i) attitudes (i.e., perceived costs and benefits); (ii) subjective norms (e.g., influence of close friends and relatives); and, (iii) perceived control over behavior (i.e., extent to which behavior is perceived as subject to control by the individual).

Using a different social-psychological approach, Miller and Pasta (1994, 1995) adopt the traits-desires-intentions-behavior framework (T-D-I-B), where fertility intentions are placed within a complex decision-making framework. Miller (2011) argues that having a child is the result of a sequence of motivational traits that translate into desires, which in turn form the fertility intention. That intention then translates into the behavior of avoiding or realizing a pregnancy. An alternative model to explain human fertility is the theory of conjunctural action (TCA), recently introduced by Morgan and Bachrach (2011). According to the TCA, fertility behavior is the result of an interaction between a unique set of social circumstances (e.g., normative expectations and structural factors) and schemas, which are mental structures that the human brain uses to represent the surrounding world and to process information. This theory differs from the TPB in that it acknowledges that fertility behavior might not only be the result of a reasoned rational deliberation, but also the result of automatic unconscious processing.

Within the existing literature, two main types of fertility intentions have been examined: (1) *quantum intentions* (i.e., intended family size); and, (2) *parity*-*progression intentions* (i.e., intentions to have a(nother) child at all or within a specific time frame). Quantum intentions have been shown to be a rather poor predictor of the total actual or realized number of children (Quesnel-Vallée and Morgan 2003), as they are subject to downward or upward adjustments over the life course (Liefbroer 2009; Iacovou and Tavares 2011). The main factors causing variations in fertility intentions appear to be partner’s expectations (Iacovou and Tavares 2011), changes in partnership status, activity status and actual fertility events (Liefbroer 2009). Parity-progression intentions are instead considered as more stable and reliable (Schoen et al. 1999; Philipov 2009), despite the fact that some studies have demonstrated a mismatch between intentions and actual behavior (Westoff and Ryder 1977; Toulemon and Testa 2005). Specifying a time frame (e.g., 2 or 3 years) has been shown to significantly improve the predictive value of fertility intentions (Billari et al. 2009; Philipov 2009).

### Partner and Partnership

Changes in partnership dynamics experienced in the past decades across advanced societies has been linked to the postponement of parenthood. A growing number of studies has shown a parallel tendency to delay union formation and parenthood (Corijn and Klijzing 2001; Mills and coworkers 2005), an increased frequency to have several partners before the first child (Wu and Schimmele 2005), and a rise in unmarried cohabitation, which has been associated with a later age at entry marriage (Bumpass et al. 1991; Mills 2004), if not a ‘retreat from marriage’ (Gibson-Davis et al. 2005).

The partner’s fertility intentions also play an important role in the realization of an individual’s intentions, since generally childbearing in advanced societies is a joint couple decision. If there is a disagreement about childbearing expectations within the couple, the positive fertility intentions of one of the partners are less likely to be realized (Thomson 1997; Schoen et al. 1999; Thomson 2002). Partnership status is also a strong predictor, with those who are not in a stable relationship being less likely to have a child (Hobcraft and Kiernan 1995; Philipov et al. 2006; Testa 2006). There has also been a growth in the decoupling of first births from marriage (Buchmann and Kriesi 2011), which is associated with an upward trend in non-marital childbearing (Dalla Zuanna 2001; Billari and Kohler 2004). Although the risk of having a first child has been shown to be lower in cohabiting versus marital unions (Brien et al. 1999; Baizán et al. 2003, 2004; Spéder and Kapitány 2009), the role of cohabitation and its relationship with childbearing compared to marriage differs across countries (Heuveline and Timberlake 2004). In France, cohabiting couples have approximately the same probability of having a child as their married counterparts (Toulemon and Testa 2005), while in the U.S., cohabitation is associated with a lower probability of childbearing (Heaton et al. 1999). Beyond the partners’ childbearing desires, a variety of other characteristics of both partners or of the couple have likewise been found to influence childbearing (e.g., Thomson et al. 1990; Corijn et al. 1996; Thomson and Hoem 1998; Jansen and Liefbroer 2006).

With the growth of more unstable relationships and higher levels of separation and divorce, another relevant topic has been the influence of union (in)stability and/or low relationship quality on childbearing. Although causal links are complex and there are important feedback mechanisms (Waite and Lillard 1991), the existing literature provides evidence for two opposing mechanisms. On the one hand, some studies find a negative relationship between low quality/instability of partnership and having children (Thornton 1978; Myers 1997). Couples experiencing marital instability are at a lower risk of having a child due to a reduced frequency of intercourse (Cohen and Sweet 1974; Thornton 1977, 1978) or because they consider the fact that children might raise dissolution costs (Lillard and Waite 1993). On the other hand, Friedman et al. (1994) argue that union instability leads to earlier childbearing since children are seen as a source of uncertainty reduction and thereby operate to enhance marital solidarity. This latter argument has also been supported by additional empirical studies (Wu 1996; Myers 1997). Rijken and Thomson (2011) find a non-linear relationship between relationship quality and fertility: women who experience a medium-quality relationship are the most likely to have a(nother) child, because they are the ones that are the most eager to invest in their relationship. Rijken and Liefbroer (2009) also investigate the impact of partnership quality on the timing of childbearing. Once again, two alternative mechanisms were isolated: the first is that a high-quality relationship offers a ‘favorable environment’ to raise children and second, that having a child may be means of ‘revitalization’ of one’s relationship.

### Gendered Division of Labor

Another important factor influencing fertility is the gendered division of domestic labor of couples within the household. Contemporary work builds on McDonald’s (2000a, b) gendered fertility theory, which argues that very low fertility is the result of a hiatus of sustained gender inequity in family-oriented social institutions. Esping-Andersen (2009), also drawing on the work by the economist Goldin (2006), sees low fertility as a consequence of the “incompleteness” of the revolution that transformed women’s roles. Empirical studies that examine gender equity at the micro-level provide interesting insights on how the gender role-set within the family affect an individual’s probability to have a child. In a qualitative study of women’s fertility in Canada, Matthews (1999) reports that women responded to feeling overburdened at home by having fewer children. Using U.S. data, Miller Short and Torr (2004) find a U-shaped relationship between gender equity within the couple and fertility: the probability of having a second child is higher in families with either very low or very high gender equality. Tazi-Preve et al. (2004) demonstrates that the unequal distribution of household labor lowered men’s fertility intentions in Austria. This concurs with the work of Oláh (2003), who in a comparison of Sweden and Hungary finds that a more equal gender division in household tasks accelerates the transition to the second child, noting that specific policies in Sweden supported this transition. In a study on Italy and Spain, Cooke (2009) finds that increases in employment equity between partners increased equity in the division of household labor, which had beneficial effects on the progression to a second child. The effects, however, differed across countries. In a comparative study of the Netherlands and Italy, Mills et al. (2008) find that an unequal division of household labor significantly impacts women’s fertility intentions when they already have a heavy load (more work hours, children), which is particularly salient for working women in Italy. Begall and Mills (2011) also demonstrate that the degree of work–family conflict plays an important role for women across many European countries, with the prevalence of part-time work and higher perceived control over work significantly predicting the intention to become a mother.

### Stepfamily Fertility

The increase in unstable and multiple unions has also brought a growth in the study of *stepfamily fertility*. This body of research demonstrates that partners who already have children from previous unions are more likely to have a child together, often considered as a union commitment effect (e.g., Vikat et al. 1999; Buber and Prskawetz 2000; Stewart 2002; Thomson 2002; Prskawetz et al. 2003). Jefferies et al. (2000) for instance, find that among British women, almost half of those who experienced a marital dissolution subsequently experience a conception within twelve months, with age of the woman and age of her youngest child being the most important factors, together with repartnering. Repartnering might therefore further fuel higher fertility quantum. Given that one child is enough to indicate commitment in a partnership, multiple relationships and subsequent partnerships might significantly contribute to total fertility.

### Income, Education and Human Capital

Socio-economic circumstances of individuals have also been studied as determinants of fertility quantum and timing. Income (and wages in particular) has attracted considerable research interest in economics. Depending on the economic model that is adopted, the effect might be different. The family economics approach, pioneered by Becker (1960), maintains that individuals obtain direct pleasure from having and raising children, and from their well-being. Children, and possibly their quality level, thus resemble a consumption good in the utility function of their parents. While the initial formulation of this theory implies a positive link between income and number of children, the large body of literature that followed focused on a *negative* relationship between income and fertility, emphasizing two aspects (see Jones et al. 2011). A first approach focuses on the *quality*-*quantity tradeoff*, proposed by Becker and Lewis (1973) and Willis (1973) (see also Becker et al. 1990). Here the argument is that an increase in income may lead to fewer children. This attributed to the fact that parents with a higher-income value children’s quality, but a focus on higher-quality raises the cost of having (and raising) children, thereby potentially reducing fertility levels. Lee and Mason (2010) apply this model to show that as income increases, lower fertility is associated with an increased expenditure in children’s human capital.

A second approach focuses on the *opportunity cost* of having children, especially for women. Since raising children requires parental (and especially maternal) time, fertility is more costly for higher-income mothers, who are therefore expected to have fewer children (e.g., Kravdal 1992). This is related to the literature that demonstrates a ‘motherhood wage penalty’, with postponement providing considerable earnings returns for higher educated women or those in professional occupations (Van Bavel 2010; Begall and Mills 2012). Miller (2010) demonstrated for example, that a year of delayed motherhood increased women’s earnings by 9 %, their work experience by 6 % and average wage rates by 3 %. Others have extended Becker’s static model by setting up dynamic economic models of the optimal timing of first birth (mostly focusing on women), based on the minimization of opportunity costs of childbearing, ‘wage penalty’ and income loss (Happel et al. 1984; Cigno and Ermisch 1989). They theorized and demonstrated that the higher the accumulation of human capital during education or the higher the returns to education, the later the transition to motherhood. Gustafsson (2001, 2002) demonstrated that women’s career planning was the main explanation for postponement, a finding replicated in more recent studies in Sweden (Gustafsson 2005), the U.K. (Kneale and Joshi 2008), Ireland (O’Donoghue et al. 2011), the U.S. (Amuedo-Dorantes and Kimmel 2005; Miller 2010) and Italy (Rondinelli et al. 2010).

A similar approach has been adopted by the rich body literature that focuses on the relationship between education and labor market trajectories and the timing of first birth. The argument is that due to the accumulation of human capital, women with higher levels of education are more likely to pursue careers and increase their earning power. This likewise releases them from the pressure to get married and have a child for economic reasons. As the opportunity costs of childbearing and childrearing increase with human capital, highly educated women are more likely to postpone marriage and births. An important critique of this approach has been put forth by Oppenheimer (1994), who argues that highly educated women are more likely to find partners who are highly educated as well. This in turn operates as an incentive for women (because they can further pool economic resources) to enter into a union and subsequently have children once they complete their education. In line with Oppenheimer’s approach, other studies find that the higher educated are more likely to have a(nother) child or have overall, higher fertility (Mencarini and Tanturri 2006; Mills et al. 2008), since: (i) they are also likely have a partner with higher education and therefore a higher wage (Behrman and Rosenzweig 2002); (ii) they have stronger bargaining power within the couple, leading to a more equal division of domestic labor; and, (iii) they can outsource housework. Although higher educated women have their first child later than their lower educated counterparts, some studies (Sobotka 2004; Kravdal and Rindfuss 2008) have highlighted that the higher educated are also more likely to recuperate at a later age. Or in other words, that the cumulative impact of late motherhood on higher-order birth rates (i.e., second or third births) disappears.

The results linking education to fertility are, however, mixed, with recent empirical results instead showing a non-relevant association between education and fertility (e.g., Skirbekk 2008). Using a natural experiment approach on school entry policies in California and Texas (which should be able to unravel causality), McCrary and Royer (2011) find that education does not significantly impact fertility. Several studies also find a strong inverse relationship between educational attainment and the timing of first births in different countries (Rindfuss et al. 1980, 1996; Martin 2000 for the U.S.; Joshi 2002 for U.K.; Lappegard 2002 for Norway; Meron and Widmer 2002 for France; Noguera et al. 2003 for Spain).

Other researchers have instead focused on the importance of educational enrolment, as opposed to the highest achieved level of education. Here findings show that individuals who are still enrolled in education are at a lower risk of having a child, likely attributed to the presence of a ‘sequencing norm’ of first finishing education, followed by parenthood (Hoem 1986 for Sweden; Goldscheider and Waite 1986 for the U.S.; Blossfeld and Huinink 1991 for Germany; Kravdal 1994, for Norway). Others have extended this research to also examine the importance of the educational field of study in relation to either socialization or self-selection effects into later occupations, which in turn impact fertility (e.g., Hoem et al. 2006; van Bavel 2010; Begall and Mills 2012). Almost all studies focus exclusively on women, with some noticeable exceptions. Winkler-Dworak and Toulemon (2007), for instance, explicitly study the convergence in explanatory factors explaining the age at first birth for women and men.

### Economic and Employment Uncertainty

Further, mostly sociological studies focus on the importance of employment status and particularly economic uncertainty on fertility outcomes. Theories of (largely economic) uncertainty are reminiscent of Easterlin’s (1976) theory of economic deprivation, which posits that in historical periods of general economic uncertainty and rising unemployment, the tendency to marry and have children appears to diminish. This also relates to Oppenheimer’s (1988, 2003; Oppenheimer et al. 1997) work on the impact of uncertainty in social and economic roles on the timing of family transitions. An increasing number of studies link economic uncertainty—often in the form of unemployment and precarious labor market situations—to the postponement of parenthood. In order to empirically measure the impact of uncertainty on the entry into parenthood in a cross-national context, Mills and Blossfeld (2005) developed a schema consisting of three types of uncertainty: economic, temporal, and employment relation. They found that under conditions of economic uncertainty, which is the caliber of economic precariousness of an individuals’ employment circumstances (e.g., lower earnings, unemployment), youth deferred long-term binding commitments such as parenthood that require a secure economic basis (Oppenheimer 1988) or what Rindfuss and Vandenheuvel (1990) refer to as the ‘affordability clause’ to have a child. Following Breen (1997), temporal uncertainty (i.e., often in the form of temporary or fixed-term contracts) reduced youth’s ability to make long-term commitments such as parenthood. Finally, lower employment relationship uncertainty (e.g., dependent workers versus self-employed or contract workers) were impeded by their more precarious positions. The impact of uncertainty, however, was highly filtered by national-level institutions, such as the amount of protection young adults received from the welfare state to shelter them from uncertainty, and gender systems, which resulted in differential responses to uncertainty of women across different national contexts (Mills and coworkers 2005).

Kreyenfeld (2010) finds that both objective economic uncertainty (unemployment) and subjective uncertainty (fear of economic situation and job security) have little impact on the postponement of parenthood, with the level of education operating as the underlying driver of the process. In other words, lower educated mothers respond to economic uncertainty by adopting the role of mothers, while their highly educated counterparts postpone childbearing.

### Fertility Preferences

An individual’s fertility decisions are shaped by his or her own *preferences*, which several authors emphasize, are shaped early in an individual’s life. Catherine Hakim’s *Preference Theory* (2003) positions the heterogeneity of women’s lifestyle preferences at the heart of fertility (and labor market) choices in advanced societies. Hakim assumes that lifestyle preferences are rather constant across the life course, with three main types: career-oriented, family-oriented and those oriented towards combining work and family. These lifestyle preferences are seen as the main driver, with policies required that take into account this heterogeneity. In a comparative study within Europe, Vitali et al. (2009) find that family-oriented women are the most fertile, whereas work-oriented women usually have fewer children or even no children at all (albeit the causal direction remains unclear). Other studies (Mencarini and Tanturri 2006; Agrillo and Nelini 2008) find that, among other factors, preferences play a crucial role in the decision to remain voluntarily childless or ‘child-free’. While Agrillo and Nelini (2008) provide a detailed overview of the psychological and sociological factors associated with voluntary childlessness, medical research often focuses on physical causes related to infertility.

Research on preferences for the sex composition of children shows an effect of sex preferences on the probability to have more children, albeit with considerable variation across countries (Hank and Kohler 2000). Andersson et al. (2006a, b) and Mills and Begall (2010), for instance, find the presence of a mixed-sex preference (i.e., preference to have at least one boy and one girl), which prompts a significantly higher likelihood to the progression to the third child to reach this goal.

### Intergenerational Transmission of Values and Behavior

The similarities of fertility histories across successive generations has also been a core area of research, focusing mainly on the stable result of a positive correlation between the number of siblings and number of own children (e.g., Berent 1953; Duncan et al. 1965; Johnson and Stokes 1976; Zimmer and Fulton 1980; Thornton 1980; Anderton et al. 1987; Axinn et al. 1994; Murphy and Wang 2001) or between the age at first birth of parents and that of their children (Rijken and Liefbroer 2009). The majority of studies concerning tempo focus on teenage motherhood, demonstrating that having had a young mother increases the risk of having a child at a young age (Furstenberg et al. 1990; Horwitz et al. 1991; Kahn and Anderson 1992; Manlove 1997). Barber (2000, 2001) and Steenhof and Liefbroer (2008) also find corresponding results for later ages and for men. Within this body of literature, the intergenerational transmission of behavior is considered to be driven by intra-familial socialization processes that occur during childhood and adolescence (Hendershot 1969; Thornton 1980; Axinn et al. 1994; Murphy and Wang 2001). The assumption is that parents transmit family values, preferences and attitudes, as well as contraceptive knowledge. Rijken and Liefbroer (2009), however, show that this effect is fully mediated by the child’s own degree of religiosity. Intergenerationally transmitted knowledge, attitudes and values can be seen as individual characteristics that have a long-term effect in the same way that genetic heritage is transmitted from parents to children.

### Biodemography of Fertility

Next to socialization mechanisms, biological and genetic factors have also been used to explain intergenerational similarities in fertility preferences and behavior (Wachter and Bulatao 2003). A series of studies have linked biological and genetic components to fertility behavior (Kohler et al. 1999; 2002a, b; Kohler and Rodgers 2003; Rodgers et al. 2008). This body of research focuses on studying the interplay between fertility, environment and genetic make-up of individuals and demonstrates that differences in the genetic composition of individuals affect their fertility outcomes and fertility related behavior. They often adopt a ‘twin design’ which compares monozygotic twins (with virtually identical genetic makeup) with dizygotic twins. This permits the separation of what proportion of the variance is attributed to genetic, shared-environment (i.e., growing up in the same household, environment) or non-shared-environment (i.e., all other factors such having different partners). Kohler et al. (1999), for example, used Danish twin data to disentangle genetic and social influences on the patterns of heritability for the number of children, finding that genetic influences appeared to largely override previous shared social (familial) environments for younger cohorts.

These types of studies are part of the emerging field of the biodemography of fertility, which is an interdisciplinary area of fertility research that combines theories from the social sciences (sociology, economics) with approaches from behavioral and molecular genetics, neuro-endocrinology, and evolutionary theory (Wachter and Bulatao 2003). The central premise is that genetic and biological dispositions of individuals influence fertility either directly via genetically mediated variations or, since many aspects regulating fertility possess considerable volitional control (e.g., decision of age at first birth, fertility preferences), via underlying temperament or personality influences on fertility decisions (Jokela et al. 2009). There is also growing evidence that genetic variance changes over time and across educational levels, meaning that the importance of social norms and individual decision-making change across time (Kohler et al. 1999; 2002a, b; Kohler and Rodgers 2003). The biology of fertility has also been revolutionized by the rapid diffusion of various types of assisted reproductive technologies (ART). ART not only provides new opportunities to extend the reproductive window for couples who desire to have children at a later age, but also enables parenthood for many couples that would have previously been considered sterile, with ART utilization increasing rapidly across Europe (de Mouzon et al. 2010).

### Socioeconomic Status and Cultural Context of Family of Origin

A related stream of research investigates the effect of the socio-economic and cultural context of the family of origin on an individual’s age at childbearing and fertility quantum decisions. Some studies have shown that there is a negative relationship between parents’ educational level (especially father’s education) and age at first birth (Michael and Tuma 1985; Blossfeld and Huinink 1991; Billari 2001a, b) as well as the number of children (Murphy and Wang 2001; Rijken and Liefbroer 2009). A negative relationship has also been found between parents’ financial situation (i.e., job status) and (expected) number of children (Thornton 1980, Murphy and Wang 2001) and age at first birth (Rijken and Liefbroer 2009). These findings suggest that in higher educated and high-status families, other goals beyond family formation are more easily transmitted, together with aspirations for material goods (Easterlin 1969; Pampel and Peters 1995). Therefore, if consumption aspirations are high, parenthood will be reduced or at least postponed. This is in line with findings that show a negative effect of employed mothers (compared to non-employed ones) on the age of first birth of their offspring (Barber 2000). For the opposite reason, parents’ religiosity is positively associated with their children’s fertility (Rijken and Liefbroer 2009).

### Reverse Causality at the Micro-Level

Several articles have explicitly attempted to uncover causality in the relationship between partnership and childbearing. Using simultaneous hazard models on U.S. data, Brien et al. (1999) show that common factors exist, with being in a partnership resulting in a higher likelihood of childbearing. The comparison between cohabitation and marriage, with similar approaches based on simultaneous hazard equations, is the focus of Baizán et al. (2003 for Spain; 2004 for Sweden and Germany), Le Goff (2002) for France and West Germany, Steele et al. (2005, 2006) and Aassve et al. (2007) for Britain. Spéder and Kapitány (2009), instead, use time-dependent fertility intentions (i.e., intention to have a child within 3 years) and look at the realization of these intentions to investigate which micro-level factors are associated with a higher probability of postponing the realization of childbearing intention beyond the planned time span.

Just as the relationship between partnership and fertility, the relationship between education and the timing of childbearing can be spurious (i.e., affected by common observed or unobserved factors), reversed, or the result of an individual’s simultaneous choice in the two life spheres. Therefore, in order to properly estimate the effect of education (or job career, or education field) on the age at first birth, potential endogeneity issues must be taken into account (Billari and Philipov 2004).

Another topic where causality is a key challenge is studying the relationship between childbearing and well-being. A limited, but growing body of research has highlighted the role of happiness as a determinant of fertility, with consistent results for both sexes. Using the European Social Survey, Billari (2009) demonstrates that happier people are more likely to intend to have a(nother) child, while using longitudinal data from Australia, Parr (2010), finds that life satisfaction is a determinant of fertility.

More attention has been devoted to investigating the opposite direction of the relationship between childbearing and well-being. While older studies seem to imply that in advanced societies children were detrimental to marital well-being (see the review of McLanahan and Adams 1987), more recent research connect childbearing decisions with well-being, in various other ways. The ‘value of children’ theory (Hoffman and Hoffman 1973; Hoffman and Manis 1979; Friedman et al. 1994), recently reconceptualized as a special case of the general theory of social production functions (see, e.g., Nauck 2007), envisions having children as instrumental in maximizing individual utility as expressed by the combination of physical well-being and social esteem. In this sense, having children when fertility control is available positively contributes to individual well-being. Building on this approach, Kohler et al. (2005) demonstrate that becoming a parent contributes positively to parents’ *happiness*. Using Danish twin data and a rigorous econometric approach that accounts for possible sources of endogeneity (e.g., genetic factors), the authors find a non-linear effect of children on happiness, especially for women. Women’s happiness increases after the first child, but having higher-order children is not associated with further increases in well-being. In Europe, parents have been found to be generally happier than non-parents (Aassve et al. 2011). Examining the dynamic relationship between having children and happiness, Pouwels (2011) finds an inverse U-shaped effect of first birth, by showing that in the year before and after the first childbirth, parents experience a sharp increase in the level of happiness. Happiness, however, appears to drop some months after the delivery and new parents are found to be unhappier than previously for a considerable period. Margolis and Myrskyla (2011) also investigate the age- and context-specificity of the relationship between happiness and childbearing.

Finally, another emerging body of research where reverse causality is relevant is the link between the field of education and age at first birth (Lappegard 2002; Lappegård and Rønsen 2005 for Norway; Martin-Garcia and Baizan 2006 for Spain; Neyer and Hoem 2008 for Austria; McDonald and Kippen 2009 for Australia; Van Bavel 2010, several countries; Begall and Mills 2012 for the Netherlands). These studies show earlier fertility among women in educational fields related to the more ‘feminine’ fields of caring (e.g., teaching, health), although there are no clear causal relationships. The mechanism is that women either self-select themselves into educational paths that lead to jobs where they are more able to combine motherhood and employment or, the difficulty of combing career and children varies by chosen career type.

## Meso-Level Determinants of Fertility

In recent years, increasing attention has been devoted to developing and applying theories that take into account the fact that individuals are positioned as social actors who make decisions and act while embedded in a web of social relationships with kin and peers. While some studies focus on the role of interpersonal interactions in shaping an individual’s fertility decision-making, others investigate how the place of residence is associated with reproductive choices. Finally, another body of research looks at the social network as a source of social capital in the form of emotional and material aid. The acknowledgement of the importance of the social network in explaining observed fertility patterns has not as of yet, however, been coupled with a convincing body of empirical research. The central reason rests with the lack of suitable data and the difficulty to model and properly identify social interaction effects and disentangle them from selection and contextual factors.

### Social Interaction

The impact of social interaction on fertility choices has received considerable attention (e.g., Bongaarts and Watkins 1996; Entwisle et al. 1996; Montgomery and Casterline 1996; Kohler 1997, 2001; Bernardi 2003). This literature has predominately focused on the diffusion of contraceptive methods in developing countries (Kohler et al. 2001) and identified two channels via which social interaction takes place. The first is *social learning*, or how individuals gain knowledge from others, and the second is *social influence*, which is how consensus in peer groups constrains attitudes and behavior (Montgomery and Casterline^1996^; Kohler et al. 2001).

In advanced societies, the evidence largely comes from small-scale qualitative studies, which illustrate that personal network contacts such as coworkers or friends are pivotal in shaping both fertility timing as well as quantum decisions (Bernardi 2003; Bernardi et al. 2007; Keim et al. 2009; Rossier and Bernardi 2009; Bernardi and White 2009). Individuals look to coworkers, for example, as a key source of social learning, to see how and whether they successfully navigate the combination of having children with a career.

Rigorous quantitative studies, however, are still lacking. To our knowledge, only a handful of studies have adopted a quantitative approach. Axinn et al. (1994) showed that the higher the number of nephews and nieces, the higher the preferred number of children. Manski and Mayshar (2003) interpret the peculiarity of Israeli’s fertility transition in light of social interactions. Billari et al. (2009), integrating a network-based approach into the Theory of Planned Behavior, find that social pressure from relevant others significantly influences women’s intention to have a child. Balbo and Mills (2011) consistently find that social pressure from kin and friends to have another child is associated with positive fertility intentions to have a second or third child. Turning to the timing of childbearing, the few quantitative studies that investigate the impact of social interaction demonstrate that when a sibling (Kuziemko 2006; Lyngstad and Prskawetz 2010) or a co-worker (Hensvik and Nilsson 2010) have a child, the risk for the individual to have a child also increases. These studies have adopted event history analysis techniques that uncover a short-term, U-shaped effect, with the contagion effect very strong and increasing in the 12th (Lyngstad and Prskawetz 2010) or 24th month (Kuziemko 2006; Hensvik and Nilsson 2010) after the relevant other’s childbearing. It then declines, becoming negligible in the long run.

An innovative approach is the one adopted by Aparicio Diaz et al. (2011), who apply an agent-based simulation model to study the effect of social interactions on the transition to parenthood in Austria during the period 1984-2004. Their simulations showed that social interdependencies among individuals can explain the substantial shift of first birth to a later age that occurred in Austria in the past decades.

### Place of Residence

Several studies document fertility differences by place of residence along several lines. First, fertility continues to vary across regions (Hank 2001, 2002; Caltabiano 2008; Kertzer et al. 2009). According to Kulu (2011), differences in desired family size explain fertility differentials between small towns and rural areas on the one hand, and urban areas on the other. Second, within urban areas, suburbs are consistently found to have higher fertility (Kulu et al. 2009), with single-family households associated with higher fertility (Kulu and Vikat 2007). These differentials persist when controlling for the socio-economic composition of areas (Kulu and Boyle 2009), suggesting that contextual effects shape fertility decisions. It is interesting to note that differences in urban and rural fertility quantum persist over time (Kulu et al. 2009), whereas differences in fertility timing have only recently emerged. As a result, postponement has been more pronounced in larger rather than in smaller settlements.

### Social Capital

The body of research focusing on the social network as a source of *social capital* (Bühler and Philipov 2005; Philipov et al. 2006) stems largely from sociological theory (Lin et al. 2001; Flap and Völker 2004; Mandemakers and Dykstra 2008). Social capital is defined as the resources that individuals have access to via personal relationships and can include goods, as well as information, money, capacity to work, influence, power or active help (Granovetter 1973; Bourdieu 1986; Coleman 1988). Building on previous sociological applications (Granovetter 1973; Bourdieu 1986; Lin et al. 2001; Flap and Völker 2004), some demographic studies have demonstrated how social capital (or the lack thereof), next to economic and cultural resources, shapes fertility decision-making (e.g., Schoen et al. 1997, Astone et al. 1999; Bühler and Philipov 2005; Philipov et al. 2006). This research looks at long-term, stable and trusting relationships (e.g., family members, grandparents, close friends or relatives) that can provide the individual or the couple with fertility-relevant supportive resources. Therefore, although the exchange of these resources occurs only ad hoc, that is, whenever they are needed (e.g., use of informal childcare when children are young), the set of relationships from which they are drawn is likely to be stable over the life course.

The majority of existing studies on social capital as a determinant of (low) fertility has focused on ex-communist Eastern European countries. This region has undergone a period of extreme socio-economic uncertainty after the end of the Soviet Union. Bühler and Philipov (2005) provide an extensive theoretical discussion of how social capital is related to social networks and how it affects the formation of fertility intentions. They also show that, in such a context, supportive network relationships and resources play a crucial role in an individual’s fertility decision-making. Consistent with this argument, other empirical studies demonstrate that the greater the social capital, the higher the probability to have (or want) a(nother) child (Philipov 2002 for Russia; Philipov et al. 2006 for Bulgaria and Hungary; Bühler and Fratzcak 2007 for Poland) and to have the child sooner (Bühler and Philipov 2005; Philipov et al. 2006).

Bühler and Fratzcak (2007) find a positive effect of social capital on fertility, with this influence being highly parity-specific and particularly strong for second births. As far as Western European countries are concerned, Hank and Kreyenfeld (2003) find that in West Germany, access to informal care arrangements (i.e., care provided by grandparents) increases the risk of first birth. Balbo and Mills (2011) show a non-linear relationship (i.e., inverse U-shaped) between informal childcare and the probability for German men of wanting a second or third child. Receiving no support at all and receiving support from too many sources (i.e., several different people) are both associated with a lower probability of intending to have another child, with the latter likely related to coordination problems.

The U.S. literature focuses on the support that kin provide to mothers, including childcare and help with raising children, especially for single mothers—here kin are seen as providing ‘safety nets’. This is particularly significant given the high rates of partnership instability and single parenthood (Swartz 2009) and is a strong focus of the ‘Fragile Families’ study (Harknett et al. 2001). Harknett and Knab (2007) find that multipartnered fertility, through the difficulties of maintaining kin networks, lowers the financial, housing and child-care support to mothers. Harknett and Hartnett (2011) likewise find that support from kin and friends are more often available to mothers who can reciprocate, and families with more difficulties have lower support.

### Confounding Factors and Reverse Causality at the Meso-Level

The acknowledgement of the importance of social interaction in explaining observed fertility patterns has not yet, however, been coupled with a convincing body of empirical research. The central reason rests with the lack of suitable data and the difficulty to model and properly identify social interaction effects (Manski 1993, 1995). The social context or other individual-level factors common among individuals can also explain similarities in behavior (e.g., same timing of childbearing) (Christakis and Fowler 2007; Cohen-Cole and Fletcher 2008; Bramoullé et al. 2009; Steglich et al. 2010; Fletcher 2011), and the social network may be chosen according to desired behaviors and changes over time.

Finally, it is essential to note that the association between the place of residence and fertility choices raises causality concerns, since the relation might be spurious and the effect can work in both directions.

## Macro-Level Determinants of Fertility

A vast literature focuses on how the cultural and institutional setting in which individuals and couples are embedded affects their fertility decision-making. Here an ‘economy versus culture’ dichotomy can be identified (Billari 2004). Whereas several studies investigate how economic trends, social policies, institutional constraints and welfare regimes influence fertility tempo and quantum, other contributions focus on the influence of values, attitudes and culture in reproductive behavior. Moreover, next to these two broad approaches, other macro-level studies look at the role of contraceptive technologies in fertility dynamics. Once again, the main challenge of this literature rests on how to deal with endogeneity and causality issues, which are elaborated upon at the end of this section.

### Economic Trends

Period effects of economic trends on fertility are usually investigated by linking the TFR to macroeconomic indicators (e.g., GDP and unemployment rate). As mentioned in the introduction, despite the fact that the influence of the timing of fertility on the TFR is mainly a measure of fertility quantum, it does incorporate timing aspects as well. The relationship between TFR and GDP is ambiguous, as Sobotka et al. (2011) demonstrated. Using data from 26 OECD countries for the period 1971–2008, they report a weak correlation between changes in GDP and period TFR, arguing that this might be a result of cross-country differences. Several studies find a pro-cyclical relationship between economic growth and fertility in the developed world. Martin (2004), for example, finds that a higher GDP is associated with higher fertility in Australia. Fertility decline during economic recessions are seen as a result of childbearing postponement, especially of first births, which can later be largely compensated during times of economic prosperity (Neels 2010). Similar arguments can be found in Kohler et al. (2002a, b); Mills and Blossfeld (2005) and Sobotka et al. (2010), who maintain that an economic downturn brings about uncertainties that in turn, lead to postponement. Some empirical studies also support this hypothesis. Santow and Bracher (2001) find a negative effect of the GDP decline on first birth rates in Sweden. Sobotka et al. (2011) show that the recent economic crisis that occurred in 2008–2009 in Europe and the U.S. seems to be associated with a decline in fertility, likely due to postponement effects.

Other studies, however, find contrasting results. Butz and Ward (1979) suggest that economic upswings bring about the increased employment of women, making children more expensive during times of economic prosperity. Therefore fertility trends are likely to be counter-cyclical. Macunovich (1996) finds evidence for this expectation in the U.S. The negative relationship between economic growth and postponement also seems to be contradicted by some recent studies. Billingsley (2010) finds that the GDP in Eastern Europe after 1990 is positively correlated with fertility postponement, a result also observed in Hungary for the timing of first birth (Aassve et al. 2006). These latter studies are examples of a broader literature that adopts the economic crisis argument to explain the sharp decline in fertility that Central and Eastern European countries have undergone after the fall of communism. Once again, however, the evidence is mixed. Kohler and Kohler (2002), using Russian data, find, for instance, a negative association between a drop in GDP and TFR at the macro-level, but this finding is not observed at the micro-level.

A related approach that has recently received great attention is the one that explains fertility patterns and cross-country fertility differences in terms of socio-economic development. Myrskylä et al. (2009) provide evidence for a fundamental change in the well-established negative relationship between fertility and development (Bryant 2007). They find that while low and medium levels of the human development index (HDI) are associated with persistent low fertility, higher HDI levels seem to promote fertility, reversing the declining pattern.

Instead of looking at GDP, other researchers maintain that indicators of consumer confidence are a better measure of economic recessions, because they reflect the subjective perception of crisis. Van Giersbergen and de Beer (1997) and Fokkema et al. (2008) find a positive relationship between this indicator and TFR in The Netherlands.

### (Un)employment Trends

Economic uncertainty has also been studied by examining the effects of *unemployment* trends on the TFR. Findings consistently showed a negative association: the higher the unemployment, the lower the quantum of fertility (Macunovich 1996; Adserà 2004; Örsal and Goldstein 2010) or the higher the postponement, which was found for first and second births (Adserà 2010, 2011).

Adopting a complementary approach, other studies focus on the relationship between female labor force participation (LFP) and TFR, showing that in OECD countries, this association has changed from negative (where countries with higher LFP had lower TFR) to positive during the 1980s. Benjamin (2001), Pampel (2001), Ahn and Mira (2002), and Kögel (2004) provide some theoretical explanations and empirical evidence to describe the change in this cross-country correlation. It is, however, challenging to assess whether this implies a change in the causal relationship between the two variables. Mishra et al. (2010), engaging in a macro-econometric analysis aimed at ruling out endogeneity in order to unravel causation, find that causality runs from changes in fertility (TFR) to changes in LFP.

Another approach on the effects of economic trends has been developed by Easterlin (1961, 1968). According to Easterlin, cyclical changes in fertility are mainly due to fluctuations in birth rates and cohort size. Members of larger cohorts face more competition and thereby reduced economic opportunities, leading to lower fertility (for further details, see review of Pampel and Peters 1995).

### Policy Measures

A second stream of research has studied the impact of policy measures (e.g., labor market, fiscal, family, or housing policies) on the timing of parenthood, as well as on fertility quantum. There is mixed evidence regarding the effectiveness of social policies on fertility (Neyer 2003; Gauthier 2007; Hoem 2008; Mills et al. 2011). Gauthier (2007) argues that their effects, although small, seem to affect the timing of fertility rather than the number of children.

A large number of studies investigate the effects of childcare provision on fertility. Most of the empirical research shows mixed findings. While some studies find that regions with poor childcare coverage have higher fertility (e.g., Kravdal 1996; Rosen 2004), others, arguing that they take endogeneity into account, find that public availability of childcare has a positive effect on fertility (Del Boca 2002; Rindfuss et al. 2010). Manuelli and Seshadri (2009) present a model and empirical analyses in which higher tax rates determine low fertility. Social security systems, and the reform in social security, have been discussed as determinants of fertility (Cigno and Rosati 1992). The relevance of social security for fertility choices is also linked to the idea that children may provide security in old age, also in advanced societies (Rendall and Bahchieva 1998; Mills and Begall 2010). Galasso et al. (2009), for instance, show that the generosity of public pensions is negatively associated with fertility.

Similar to economic factors, with which they are closely interrelated (Hoem and Hoem 1997), the effect of family policies varies according to the institutional context and individual-level determinants. For this reason, there has been only minor attention to pure macro analyses, focusing on time-series variation within a country. This includes an article by Ermisch (1999), who finds that generous child allowances in Britain encourage young motherhood and Hoem (2005) and Andersson et al. (2006a, b), who show that parental-leave allowance reduces postponement in Sweden. The majority of research on policy measures and timing of childbearing consists of either micro-level (individual or couple) studies, where the policy variable is one of the independent variables, or cross-national studies (sometimes multilevel) involving nations with differing policies. Although each approach has its drawbacks (for a detailed discussion see Neyer and Andersson 2008), the latter two methods permit the examination of interactions between analytical levels.

### Welfare Regimes

Building on the work of Esping-Andersen (1990, 1999), numerous scholars have explained cross-country differences in fertility and life course patterns by linking them to different institutional constellations (e.g., Mills and Blossfeld 2005; see also Neyer 2003 for a feminist critique). As described in Mills and Blossfeld (2005), who link different welfare regimes to fertility postponement, differences between welfare regimes manifest themselves in the priority of: (1) active employment-sustaining labor market policies (i.e., the commitment to full employment); (2) welfare-sustaining employment exit policies (i.e., support for those who are outside of the labor market such as youth, unemployed, ill, poor, family care workers, pensioners); (3) the scope and generosity of family allowances and services (i.e., maternity/paternity leave, childcare); and, (4) the share of the public sector in the labor force. This constellation of policies in turn impacts the safety net that individuals can draw upon if they are unemployed, employment regulations and family-related services (childcare, leave), which in turn enable or constrain decision-making about entry into parenthood or having additional children.

Defamilialized regimes, where the households’ welfare and caring responsibilities are largely supported by the welfare state (and not the family), such as Nordic socio-democratic countries, or market provision (Anglo-Saxon liberal market regimes), enable higher fertility. Conversely, familistic states (Conservative and especially Southern European regimes), where the majority of the economic and caring responsibilities rest on the family, where institutions also support a traditional division of the domestic labor (i.e., the so-called male-breadwinner model; Blossfeld and Drobnič 2001), constrain fertility, resulting in lower fertility levels.

This literature is mainly developed at the theoretical level. Researchers focus on the different manifestations of lack of state support, describing how these factors make it difficult to combine work and family, especially for women, forcing them to choose between a career versus motherhood, thereby resulting in postponing or forgoing of children (e.g., Castles and Ferrera 1996; Mayer 2004). The direct empirical research linking specific welfare regimes explicitly to fertility is limited due to the high complexity of modeling these regimes and, similar to research on social policies and fertility, it consists of either analyses at the micro-level where the effect of the different welfare regimes is measured by simple dummy variables, or cross-national studies (sometimes multilevel) involving countries with different institutional arrangements. Examples of this empirical body of research are reviewed in the final section of this paper (Sect. 5), where the interaction between micro- and macro-level is discussed.

### Value and Attitude Changes

This stream of research largely stems from the ‘second demographic transition’ (SDT), developed by Lesthaeghe and van de Kaa (1986) to interpret demographic changes in industrialized societies. According to this theory, ideational changes, that mainly consist of the rejection of institutional control, accentuation of individual autonomy and the rise of self-realization needs (Surkyn and Lesthaeghe 2004), are the driving forces of new family arrangements and behaviors, among which fertility postponement, reduced number of children, or childlessness, that have developed since the 1960s. The SDT framework has been used as an alternative (Lesthaeghe 1983 for Belgium) or complementary explanation (Billingsley 2010 for Eastern European countries), next to economic cycle effects, to fertility quantum variations. Lesthaeghe (2010, p. 242), one of the proponents of this theory, has however underlined that “the SDT theory fully recognizes the effects of macro-level structural changes and of micro-level economic calculus. But… the SDT theory does not consider cultural change as endogenous to any economic model, but as a necessary additional force with its own exogenous effects on demographic outcomes”.

As highlighted by van de Kaa (1997), such ideational changes may occur in different periods and at a different intensity across diverse areas. Some articles provide support for the SDT, showing a delay of fertility in relation to increased autonomy and independence, such as Liefbroer (2005) for the Netherlands and Bernhardt and Goldscheider (2006) for Sweden. An interesting approach is the developmental idealism framework elaborated by Thornton and Philipov (2009), according to which ideational influences and the intersection of these ideational influences with structural factors are the main forces driving the fertility decline in Central and Eastern Europe after the end of the Soviet Union.

Other studies focus on the impact of changing social norms on fertility. Several researchers have documented the relevance of *age deadlines* for childbearing (i.e., ages after which it is not socially acceptable to become a parent) (Settersten and Hagestad 1996; Liefbroer and Billari 2010). Billari et al. (2011) illustrate that age deadlines are positively associated with the prevalence of ART in a given country.

Similar to the welfare-regime research, this literature is also mainly theoretical. This is primarily attributed to the difficulties in collecting data on ideational changes at the societal level. To overcome this issue, some studies (e.g., Liefbroer 2005; Bernhardt and Goldscheider 2006) empirically operationalize changes in values and norms using micro-level individual measurements, although they assume the value changes to occur at the societal level.

### Historical and Cultural Continuities

Some studies identify historical and cultural continuities—or path dependency—as the roots of present fertility behaviors, reaching similar conclusions to that of Esping-Andersen (Reher 1998; Micheli 2000; Dalla Zuanna 2001). However, this literature, by strongly emphasizing the importance of cultural background, assumes that culture has shaped institutional settings (Pfau-Effinger 1999).

This body of research can be distinguished along East–West and North–South divides (see Billari 2004 for a more detailed overview). The East–West divide in Europe, running along an imaginary line connecting Trieste and St. Petersburg, was first noticed by Hajnal (1965). On the West side of the ‘Hajnal line,’ areas were characterized by late and not universal marriage, whereas to the East of the line, marriage was early and widespread. Historical continuities are then assumed to explain why birth happen earlier to the East of the Hajnal line. The North–South divide, first elaborated by Reher (1998), considers the strength of intergenerational family ties: while Southern countries are characterized by strong family ties, Northern areas generally have weak family ties. The main argument is that systems characterized by strong kinship and intergenerational relationship (e.g., Southern European familistic countries) are those where couples have lower fertility (Dalla Zuanna 2001; Livi-Bacci 2001) and young people delay the transition to adulthood, in turn implying a postponement of childbearing (Billari 2004). It is essential to note that familistic regimes, both from an institutional as well as cultural point of view, are not ‘per se’ detrimental to fertility. It is rather the interaction of these systems with the recent increased female status in the educational and labor market system and lack of institutional support to combine work and family that is the root cause of low fertility (Feyrer et al. 2008; Mills et al. 2008).

Studies on immigrants, linking fertility in the place of origin to the behavior of individuals in a ‘destination’ country have also shown the relevance of cultural continuities (Fernández and Fogli 2006, 2009), with continuity in behavior. Nevertheless, migrants often show behavior that converges to their place of destination, demonstrating that adaptation prevails on selectivity (Kulu 2005).

### Contraceptive and Reproductive Technologies

Fertility differentials at the macro-level are not only explained by ‘economy’ and ‘culture’. Researchers have widely studied the crucial role of the ‘contraceptive revolution’ on fertility quantum (e.g., Frejka 2008) and fertility postponement (for a detailed review see Sobotka 2004). The spread of modern contraception, and especially the pill, has radically changed the nature of the fertility decision-making and contributed to the reduction in the number of children and the postponement of childbearing (Goldin 2006). Murphy (1993) argues that short-term changes in fertility in England and Wales during the 1970s and the early 1980s can be better explained by the swings in *contraceptive pill use*, due to fears of the pill’s side effects. Bailey (2010) exploits variation in laws permitting the sales of contraceptives in U.S. states as a natural experiment to show that contraception causally contributed to the reduction of period fertility rates. Sobotka et al. (2010) assesses the importance of ART (Assisted Reproductive Technology) on fertility using data from Denmark. They project a rising share of children born as a result of ART, with a 5 % contribution to the TFR of the 1975 birth cohort. Moreover, the development ART seems to challenge the biological limits to postponement (Billari et al. 2007; see Leridon 2008 for an analysis of the extent to which ART affects the probability of becoming a parent at advanced ages).

### Endogeneity of Policies and Reverse Causality at the Macro-Level

Difficulties in disentangling the impact of policies from other observable or unobservable factors have often frustrated the effort to uncover policy effects on fertility. It is difficult to separate the impact of any specific policy from the broad range of policy instruments that potentially influence fertility and it is problematic to empirically establish whether a specific policy was successful due to the temporal lag between policy initiation and take-up. Finally, there is the problem of endogeneity of policies, in that they may not only impact fertility and induce change, but are often a reaction to changes in fertility and an integral feature of these changes. A rigorous analysis conducted by Kalwij (2010), however, finds a positive effect on fertility quantum due to an increased expenditure for family policy programs that help women to combine family and employment, thereby reducing the opportunity cost of children. Fiscal policies, that more easily allow implementing quasi-experimental strategies, have attracted the attention of many economists. Positive effects of fiscal incentives on fertility quantum have been found in Germany (Buttern and Lutz 1990), Sweden (Walker 1995), Canada (Milligan 2005), and the U.S. (Whittington 1993). Gauthier and Hatzius (1997) found more mixed results in employing a cross-country panel.

## Discussion

Our review demonstrates that research on fertility in advanced societies is not only extensive, but continues to thrive and evolve in innovative ways. The central goal of this paper was to evaluate the current state of fertility research in order to classify and assess different approaches and the knowledge they have added. A secondary goal was to classify existing research according to the three analytical levels of macro-, meso- and micro-level approaches and findings. We likewise placed considerable attention to causality and endogeneity issues.

We first demonstrated that there have been considerable advances on several fronts in the study of micro-level determinants. In addition to the consideration of key determinants, such as employment, income, and education (and nuances within these areas), promising new and innovative research has focused on how the gendered division of labor, family composition (e.g., stepfamilies), preferences and intergenerational transmission of values and behavior impact fertility. Although there have been recent advances in including new topics such as the biological and genetic underpinnings of fertility and new family forms, considerable challenges for future research still remain. The first is the availability and affordability of data with sufficient information such as biomarkers or genetic data, but also data that properly captures new types of family forms. Although growing, this type of data that combines genetic and social survey data remains limited. A second related issue, particularly for the introduction of serious biodemographic research, is the need to collaborate with experts and properly understand how to properly integrate this type of information and biological mechanisms in our theoretical, but also statistical models.

This review also highlighted core meso-level factors impacting fertility, including the emerging field of social interaction, social capital and networks and place of residence. Although a growing number of (primarily qualitative) studies started to address these meso-level factors, core challenges still remain. As noted previously, there is no large-scale quantitative network data that has been collected to examine how social networks impact fertility. Of the data that has been collected, the network measurements remained limited. This is partially attributed the high costs of collecting such data, but also the high respondent burden when gathering this type of information, which makes it difficult to include within an existing survey.

At the macro-level we summarized the key determinants that have been studied, ranging from economic and (un)employment trends, to policy measures, welfare regimes, value and attitude changes, historical and cultural continuities, contraceptive use and new reproductive technologies. As we noted previously, a key challenge for the credible integration of these macro-level factors for understanding fertility is the need to move from purely theoretical discussions to more convincing empirical tests of this link. Although researchers often claim that macro-level factors such as the welfare regime constellation or societal values impact fertility (and other demographic) behavior, there are few successful empirical attempts to empirically underpin these claims. At all levels we also addressed challenges related to reverse causality and confounding factors and for macro-level factors, the issue of the endogeneity of social policies and reverse causality, which will be discussed in more detail shortly.

Some more general problems of current research that we can draw from a broader reading of this review can provide opportunities in helping us to understand improvements for future research. Two problems that became apparent during this review were the clear boundaries between disciplines and geographical areas. First, research on fertility is highly multidisciplinary, i.e., researchers from several disciplines engage in explaining the timing and quantum issues surrounding fertility. However, there are limited instances of interdisciplinary research, simultaneously involving scholars from different disciplines or adopting theoretical and/or methodological approaches of different disciplines. Citation patterns are highly disciplinary-specific, with articles often ignoring clearly relevant research published from other disciplines. It is easy to say that research on fertility would highly benefit by crossing disciplinary boundaries more often, perhaps starting from reading each other’s research more often.

A second related point is the relevance of geographic boundaries. Research on fertility on Europe (mostly conducted by European scholars) and research on fertility on North-America (mostly conducted by North-American scholars), or in other words, the bulk of research on fertility, often do not communicate with one another. This was apparent during several places during our review where conflicting theories and findings were presented from North-American and European scholars. Topics, approaches (including the type of data) and again citations remain somehow separated, albeit research in Europe has clearly been fundamental in illuminating the role of macro-level factors, largely due to the often cross-national comparative approach. Not surprisingly, scholars working on other advanced areas are more successful in bridging the two continents over the Atlantic. Also, here it is easy to say that a general understanding of fertility choices would be easier by bridging the findings and approaches related to all advanced societies.

Two additional problems are related to methods, data and analytical strategy, which once again are apparent when we stand back to reflect in more general terms from this literature review. First, and related to the international character of fertility research, despite efforts of developing comparative, mostly aggregate data (such as the Human Fertility Database by the Max Planck Institute for Demographic Research and the Vienna Institute of Demography), most research focuses on micro-data that do not usually permit highly comparable research. We could therefore improve fertility research by developing comparable data collection in many countries—including very importantly, the U.S. and Japan in micro-level comparative fertility research. This is even more important given the increasing geographical mobility across countries. Only further collaborative efforts by researchers and funding agencies will enable us to uncover fundamental mechanisms operating at different levels and affecting fertility choices. Second, given that fertility can only be observed (as opposed to experimentally induced) by researchers, the issue of causation versus spurious association lingers as a major problem. Attention to causality is heterogeneous in the literature—undoubtedly with an advantage for studies arising from the tradition of economics. Causal interpretations are widespread also in studies that discover associations (which is a serious problem), but some studies clearly do not aim at understanding or studying causation (which is a lower-order problem related to how ambitious researchers and disciplines are). Further steps towards recognizing the importance of methods aimed at unraveling causality in observational data would contribute to conducting higher-quality fertility research. Researchers and policy-makers alike would gain much more by adopting a program evaluation perspective for the evaluations of policies that might affect fertility choices.

Furthermore, our review uncovered that three problems emerge concerning the actual factors studied, ranging from individuals to context. Research on men, or in other words, the fertility of men and fatherhood, remains very limited, albeit growing. It is clear that a gendered approach is necessary, but this implies that both genders should play an equal role in our understanding of fertility choices. More research including both men and women would improve our knowledge. Related to this first problem is the second problem of couples. For both theoretical and (lack of) data-related reasons, fertility choices have been investigated mostly from an individual perspective. The limited research, and data, existing on couples show the incredible value of addressing fertility as a joint decision. A third problem relates to the limited knowledge of the importance of meso-level factors. Here the theory is more developed than the actual instruments such as the collection of quantitative data having a kinship and/or network-based approach, which we addressed previously. Efforts in using innovative analytical techniques such as agent-based modeling are promising. Recent innovative designs (e.g., the Add Health study in the U.S. or the Netherlands Kinship Panel Study) also provide some insights on future directions.

Finally, promising research avenues are those emphasizing the interaction of factors located at different analytical levels. As already mentioned in the policies and welfare regime sections, studies that adopt a cross-country comparative life course approach often position nation-specific institutions as path-dependent structures that shape micro-level individuals’ characteristics and enable or inhibit the ability to have children and to have them at a particular period in their lives (i.e., during education, while remaining employed). National institutions or forces such as employment and education systems, welfare regimes, social policies, family, and gender systems are historically based and country-specific and determine the degree to which people are affected by macro-level changes (Mayer 2004). Micro-level factors, such as partnership status, might interact with the macro-level of institutions and culture. One example is the difference between cohabitation and marriage as determinants of childbearing. Baizán et al. (2004) find that in Sweden such differences are almost negligible as compared to West Germany. Comparing the 1958 and 1970 birth cohorts in the UK, Steele et al. (2006), find that the links between cohabitation and childbearing have strengthened over time because of changing cultural forces. Women may decide to postpone childbearing to avoid marriage particularly in less gender-equal societies (e.g., Japan) because they do not want to be forced into motherhood and out of employment (Rindfuss et al. 2004).

Throughout this review, we have already cited some studies that adopt a cross-country approach, showing how forces and constraints at the macro-level can impact micro-level dynamics. Kalwij (2010) and Begall and Mills (2011), for example, show how different welfare regimes and family policies can facilitate or constraint an individual’s work–family balance.

McDonald’s gender theory (2000a, 2000b) and related approaches (Chesnais 1996; Esping-Andersen 1999, 2009) are among the most relevant examples of interaction between micro-level factors (i.e., employment status and gender equity within the family) and macro-level factors, (i.e., welfare regime) (see also Cooke and Baxter 2010). McDonald claims that very low fertility occurs where and when high levels of gender equity in individual-oriented institutions, such as education and market employment, are coupled with low levels of gender equity in the family and family-oriented institutions. Put differently, if women are provided with opportunities near to equivalent to those of men in education and labor market systems, but these opportunities are then severely limited by having children because they cannot reconcile work and motherhood, then, on average, women will restrict the number of children. Although this theory has often been used in explaining low fertility, empirical applications are still lacking (see Mills 2010).

Another example of meso-macro interaction is the study of Balbo and Mills (2011). They show that social pressure and social capital are highly institutionally filtered, having a much stronger effect on an individual’s intention to have another child in familistic contexts, that leave caring responsibilities to the family and encourage a male-breadwinner model.
